# Turkish Translation, Validation, and Reliability Analysis of Pediatric Eosinophilic Esophagitis Symptom Severity Module Version 2.0

**DOI:** 10.1097/PG9.0000000000000243

**Published:** 2022-10-25

**Authors:** Ozlem Yilmaz, Nuray Kizilkan Uslu, Taner Ozgur, Dilek Guller, Gokhan Tumgor, Merve Usta, Esra Polat, Tanju Basarir Ozkan, Gokhan Baysoy, Meltem Korkut Ugras, Arzu Baygul, Cansin Sackesen, Cigdem Arikan

**Affiliations:** From the * Paediatric Allergy, Koç University School of Medicine, Istanbul, Turkey; † Paediatric Gastroenterology, Koç University Hospital, Istanbul, Turkey; ‡ Paediatric Gastroenterology, Bursa Uludag University, Bursa, Turkey; § Paediatric Gastroenterology, SBÜ Sariyer Hamidiye Etfal EA, Istanbul, Turkey; ∥ Paediatric Gastroenterology, Cukurova University Medical School, Adana, Turkey; ¶ Paediatric Gastroenterology, SBÜ Sariyer Hamidiye Etfal EA, Istanbul, Turkey; # Paediatric Gastroenterology, SBÜ Umraniye EA, Istanbul, Turkey; ** Paediatric Gastroenterology, Medipol University, Istanbul, Turkey; †† Paediatric Gastroenterology, Yeditepe University, Istanbul, Turkey; ‡‡ School of Medicine, Koç University, Istanbul, Turkey; §§ Paediatric Gastroenterology, Koç University Hospital, Istanbul, Turkey; ∥∥ Koç University Translational Medicine Research Centre, Kuttam, Istanbul, Turkey.

**Keywords:** children, eosinophilic, esophagitis, validation, reliability, symptom

## Abstract

The Paediatric Eosinophilic Esophagitis Symptom Severity Modules Version 2.0 (T-PEESv2.0) was developed in English as a valid, reliable questionnaire for follow up. This work aimed to develop a Turkish version of T-PEESv2.0 via translation and cultural adaptation and then to test its validation and reliability.

**Methods:**

The PEESv2.0 was translated into Turkish by standardized procedural steps completed in cooperation with the Mapi Research Trust. The final version of the questionnaire was submitted to eosinophilic oesophagitis patients or their parents at 2 times point separated by 1 week. An age-matched control group was used to test the discriminant validity. Construct validity was tested using the Wilcoxon test, and internal consistency was tested using Cronbach’s alpha. Test-retest reliability was measured with Cohen’s kappa and intraclass correlation coefficient.

**Results:**

One hundred twenty-eight participants (70 patients, 58 parents) were enrolled. Fifty-eight (39.1%) of them completed T-PEESv2.0-parent by proxy and 70 (54.7%) were T-PEESv2.0. The Cronbach’s alpha coefficient and intraclass correlation coefficient for test-retest reliability were >0.70 for both questionnaires and for all domain (frequency and severity) and total scores. For discriminant validity analysis, subscale (frequency and domain) and total scores of the patient group were compared with those of the control group. The subscale and total scores were significantly different between the groups (*P* < 0.05).

**Conclusion:**

T-PEESv2.0 appeared to be valid and reliable, ready to be introduced as a clinical and research tool for the assessment of patients with eosinophilic oesophagitis.

**What Is Known**

- The Paediatric Eosinophilic Esophagitis (EoE) Symptom Severity Modules Version 2.0 (T-PEESv2.0) is a noninvasive method (questionnaire) established for follow up purposes in children with EoE.
- T-PEESv2.0 is in English

**What Is New**

- First validated multi center study to translate T-PEESv2.0 into Turkish

## INTRODUCTION

Eosinophilic esophagitis (EoE) is characterized clinically by symptoms of esophageal dysfunction and histologically by eosinophil predominant inflammation localized at the esophagus (1). The symptoms of pediatric EoE vary according to the age ranging from the easily recognizable presentations such as food bolus impaction and dysphagia seen mainly in children and adolescents to the most nonspecific ones associated with feeding difficulties (including vomiting, regurgitation, and feeding refusal), which can result in failure to thrive in infants and toddlers (2). Due to the nonspecific mixed symptoms of EoE and other diseases and the adaptation behavior of the eating habits of the patients through the disease progression, it is crucial to develop and implement patient and parent proxy-reported validated symptom assessment tools to better analyze the outcomes in pediatric EoE both in clinical practice and for research purposes.

Pediatric Eosinophilic Esophagitis Symptom Severity Module Version 2.0 (PEESv2.0) is a pediatric EoE symptom tool developed from key self-reported and parent proxy-reported symptoms of the EoE identified through cognitive interviews (3). PEESv2.0 has 2 metrics, 1 report for children and teens aged 8–18 years (PEESv2.0-children and teens) and the other for parents of children and teens aged 2–18 (PEESv2.0-parent by proxy). PEESv2.0 focuses on the perceptions of pediatric EoE patients and their parents on the aspects of the frequency and severity of symptoms related to EoE (3,4,5).

PEESv2.0 was the first validated and widely accepted pediatric EoE symptom score metric, which utilized patient-related outcomes rather than subjective physician directed questions (3). A validated Turkish version of PEESv2.0 does not exist, yet. Therefore, this study aimed to develop a Turkish version of PEESv2.0 via translation and cultural adaptation and then to test its validation and reliability.

## METHODS

This was a methodological study of translation, cultural adaptation, and validation of PEESv2.0-children and teens and PEESv2.0-parent by proxy. The study was approved by the Biomedical Research Ethics Committee of Koç University (IRB2020.231.IRB2.065).

### Scaling and Scoring of PEESv2.0

PEESv2.0-children and teens and PEESv2.0-parent by proxy is a patient self- and parent proxy-report pediatric EoE symptom assessment tools, which were developed by Franciosi et al in 2011. Both metrics are composed of 20 items investigating 2 aspects: Frequency (11 items) and Severity (9 items). The frequency domain rates the impact of each item on a 5-point Likert scale ranging from “Never” to “Almost always” whereas the severity domain ranging from “Not bad at all” to “Very bad” with face emojies expressing different emotional states. Three types of scores are calculated: Item and Domain scores (Frequency and Severity) and Total metric score. The item score: The 0–4 scale items are transformed to 0–100 as follows: 0 = 0, 1 = 25, 2 = 50, 3 = 75, 4 = 100. The domain score is computed as the sum of the items over the number of items answered (mean). Total metric score is also calculated as the sum of all the items over the number of items answered on all the scales (mean). The higher the score, the worse the symptoms. If there are fewer than 50% of missing values for the items, the missing values are counted (Nmiss). Then, the item scores are summed and divided by the number of items in the scale minus Nmiss. If more than 50% of the items in the scale are missing, the Scale Score is not computed and excluded from the analysis.

### Translation and Language Validation of PEESv2.0

In the first instance of the translation process, consent was obtained from the author of PEESv2.0 and PEESv2.0-Parent by Proxy and Mapi Research Trust (ePROVIDE: Request no. 180368; https://eprovide.mapi-trust.org/). The English language version of PEESv2.0-children and teens and PEESv2.0-parent by proxy modules was translated into Turkish language in 4 steps: (1) forward translation: 2 native Turkish speakers both of whom are fluent in English, independently translated the original versions of PEESv2.0 metrics into Turkish. The results were discussed and a reconciled version was produced. (2) Backward translation: 2 independent translators, both native in English, translated the forward translations back into the source language (English) and 2 investigators (OY and NUK) compared it with the original one for conceptual equivalence. Content validity of PEESv2.0-children and teens and PEESv2.0-parent by proxy was assessed for language clarity and understandability by 5 experts in pediatric gastroenterology rating each item on a 4-point scale ranging from 1 (not relevant) to 4 (highly relevant) to see any misunderstandings or inaccuracies (Table 1). Backward translations were revised according to advice of colleagues and resent to them for re-evaluation and rescoring. (3) Cognitive interviewing: The backward translated and revised versions of questionnaires were then reviewed by 5 children and 5 parents. Any uncertainties were discussed with the coordinator (CA) by face-to-face interviews. (4) Proofreading: Items were finalized at this stage if there were no further changes recommended by either the patients or their caregivers. All stages were completed in cooperation with the Mapi Research Trust and the finalized versions (T-PEESv2.0-children and teens and T-PEESv2.0-parent by proxy) were sent to Mapi Research Trust along with the report detailing each step. A final approval was obtained from the company.

**TABLE 1.** Scoring for translated questions

Parameters	Score
The item is very sufficient to explain the content	4
The item is sufficient to explain the content but minor revision is required.	3
The item is confusing, revision is required	2
The item is irrelevant	1

### Study Participants

Children aged between 2 and 18 years and their parents being treated with a clinicopathological diagnosis of EoE (symptoms of esophageal dysfunction and histology reports of at least 1 endoscopy ≥15 eosinophils isolated to the esophagus) from the 6 different center’s Pediatric Gastroenterology Clinics were invited to participate in the study and an informed consent was obtained from children or their parents. The participants filled out T-PEESv2.0-children and teens or T-PEESv2.0-parent by proxy. All of the participants had refilled the metrics out 1-week later to examine the test-retest reliability. A control group of age-matched healthy children had also filled out the metrics to test the discriminant validity.

### Statistical Analysis

Characteristics of EoE patients and controls were presented as median with range or as mean with standard deviation according to the distribution parameters. In the analysis of statistical significance of selected characteristics of the patients, chi-square test, independent samples T-test or Mann-Whitney *U* test were used where necessary.

The Cronbach’s alpha (α) value was used to determine internal consistency of the metrics. The test-retest reliability was determined using the intraclass correlation (Spearman) between repeated measures of T-PEESv2.0-children and teens and T-PEESv2.0-parent by proxy. Discriminant validity was evaluated by independent samples T-test or Mann-Whitney *U* test to compare the patient and the control groups. All statistical analysis was performed using SPSS Statistics version 22.

## RESULTS

### Content Validity Procedure

After the forward and the backward translation process, 6 experts assessed content validity of T-PEESv2.0-children and teens and PEESv2.0-parent by proxy by rating each item on a 4-point scale (Table 1) and explained their opinions to correct the Turkish version of the items. Each item rated less than 4-point discussed with the coordinator and then revised. The revised backward translations were resent to the same experts and, at this stage, each item in the revised forward translations were scored as “4-point” (Table 2).

**TABLE 2.** The revised items during content validity procedure

Original item	Backward translation	Revised backward translation
**Both metrics**		
Frequency domain: almost never	Hemen hemen hiç	Neredeyse hiç
Severity domain: bad	Kötü	Şiddetli
**T-PEESv2.0-parent by proxy (2–18 years**)		
Question 11: How often does your child need to drink a lot to help swallow food?	Çocuğunuzun lokmasini yutarken çok sivi içme ihtiyaci ne sikliktadir?	Çocuğunuzun lokmasini yutmaya yardimci olmak için çok sivi içme ihtiyaci ne sikliktadir?
Question 19: How often does your child eat less than others?	Çocuğunuz ne siklikta diğerlerinden daha az yemek yer?	Çocuğunuz ne siklikta diğer çocuklardan daha az yemek yer?
Question 20: How often does your child need more time to eat than others?	Çocuğunuz, yemek yemek için diğerlerinden daha çok zamana ne siklikta ihtiyaç duyar?	Çocuğunuz, yemek yemek için diğer çocuklardan daha çok zamana ne siklikta ihtiyaç duyar?

### Cognitive Interviewing

A face-to-face interview with parents representing the study population was conducted on parents and children >8 years of a total of 10 respondents. At this stage, 2 respondents showed comprehension difficulty in question 17. The statement of “back up your throat” in the original version of question 17 had been translated initially as “çikarir” (means: spits up). Then, it revised as “boğaziniza geri gelir” (means: come back to your throat).

The final Turkish translations of the metrics were approved by the survey’s copyright owner (Table 2).

### Demographics of the Participants

One hundred eighty-four patients were recruited and collected. Five participants with more than 50% of the items were missing in the metrics were excluded (2 for T-PEESv2.0-parent by proxy; 3 for T-PEESv2.0-children and teens). Therefore, 179 participants were enrolled in the study. The patient and the control group were composed of 97 (54.2%) and 82 (45.8%) participants, respectively. The median age (IQR) of the patient and the control groups was 9 (6.75) and 9 (6.75) years, respectively. One hundred nine of the study cohort (60.9%) were male. One hundred twelve parents filled out T-PEESv2.0-parent by proxy and 67 patients filled out T-PEESv2.0-children and teens. Of the parents, 77.6% (n = 87) were mothers and 22.4% (n = 25) were fathers. The median (IQR) age of parents was 34.5 (6) (Table 3). The educational levels of enrolled parents were university (23%), high school (66%), and secondary school (11%) (Table 3).

**TABLE 3.** Missing values in the questionnaires

	Participants with missing items (n)	Missing items (n)	Question number of missing items
**T- PEESv2.0-parent by proxy**			
	1	8	2,4,6, 8, 10, 12, 14, 16
	1	6	4, 6, 9, 11, 12, 14
	1	4	17, 18, 19, 20
	8	1	Q2 (n = 2), Q4 (n = 2), n = 1 for Q 6, Q10, Q18, and Q20
**T- PEESv2.0**			
	1	8	2, 4 10, 12, 14, 16, 18, 16
	1	7	2, 4, 6, 8, 10, 12, 14
	1	4	17, 18, 19, 20
	2	2	3, 5, and 13, 15
	4	1	N =1 for Q1 and Q20, n = 2 for Q3

There were 20 participants (T-PEESv2.0-parent by proxy: 11; T-PEESv2.0: 9) with missing items in the completed questionnaires (Table 3). Regarding T-PEESv2.0-parent by proxy, 8 parents had 1 missing question and the remaining 3 had 4, 6, and 8 missings, respectively. Regarding T-PEESv2.0, 1 patient had 8 missing questions, 1 patient had 7 missings, and the remaining 7 patients had missings ≤4 (4 missing: 1 patient, 2 missing: 2 patient, 1 missing: 4 patients). No missing-item predilection was found. Therefore, we think that choice not to answer may be an explanation for the missing items rather than comprehensibility and difficulties encountered in compiling the T-PEES.

### Validity

A control group with similar age and sex to the patient group was generated for validity analysis of both metrics (*P* > 0.05) (Table 4).

**TABLE 4.** Comparisons between the patient and control group defined by the type of metrics

Variable median (25p–75p)	T-PEESv2.0-parent by proxy (2–18 years)			T-PEESv2.0-children and teens(8–18 years)		
	Patient	Control	*P*	Patient	Control	*P*
**n (%**)	57 (50.9)	55 (49.1)		40 (59.7)	27 (40.3)	
**Sex-male (%**)	41 (71.9)	29 (52.7)	0.04	27 (67.5)	12 (44.4)	0.06
**Age, year**	7.71 (5.1–10.6)	7.0 (5.0–12.0)	0.47	10.67 (9.18–14.43)	11.0 (9.28–15.45)	0.42
**Frequency score**	34.09 (15.34–48.29)	9.09 (3.22–22.54)	**<0.001**	25.00 (13.63–39.77)	4.54 (2.27–13.63)	**<0.001**
**Severity score**	36.11 (8.33–46.70)	7.29 (2.77–15.62)	**<0.001**	22.22 (9.72–44.44)	2.77 (2.77–9.72)	**<0.001**
**Total score**	33.75 (12.37–46.56)	7.69 (4.68–19.79)	**<0.001**	26.25 (14.37–40.87)	3.75 (2.50–10.62)	**<0.001**

For discriminant validity analysis, all domain (Frequency and Severity) and Total scores of both metrics of the patient group were compared with those of the control group. The subscale and total scores were significantly different and higher in the patient group than the control group (*P* < 0.001 for each subscale) (Table 4).

### Reliability

Ninety-seven participants with EoE and their parents were included in the reliability analysis. The Cronbach’s alpha coefficient for internal consistency was found >0.70 for both T-PEESv2.0-children and teens and T-PEESv2.0-parent by proxy. For test-retest reliability analysis, the participants refilled out the metrics 1-week later. Intraclass correlation coefficient for test-retest reliability were >0.70 for both metrics and all domain (Frequency and Severity) and total scores (Table 5).

**TABLE 5.** The scores according to questionnaires and domains and test-retest reliability results

Tools by age group	Domain scores	Mean ± SD	Cronbach’s alpha	ICC
**T-PEESv2.0-parent by proxy (2-18 years**)	Frequency*	34.63 ± 20.71	0.98	0.94
	Severity†	32.69 ± 23.33		0.96
	Total metric	33.76 ± 21.43		0.95
**T-PEESv2.0-children and teens (8-18 years**)	Frequency*	28.83 ± 19.37	0.97	0.95
	Severity†	28.47 ± 22.51		0.95
	Total metric	28.64 ± 20.13		0.95

*Questions: 1,3, 5, 7, 9, 11, 13, 15, 17, 19, 20.

†Questions: 2, 4, 6, 8, 10, 12, 14, 16, 18.

ICC = Intraclass correlation; SD = Standard deviation.

## DISCUSSION

This study demonstrates that T-PEESv2.0 which is the Turkish translated and culturally adapted version of PEESv2.0 is a valid and reliable metric ready to use in clinical practice and for research.

Eosinophilic esopahagitis is a disease with symptoms of esophageal dysfunction. The diagnostic delay in EoE may be years due to its vaguely recognizable symptoms, especially in the pediatric population (7). Therefore, a pediatric symptom metric such as PEESv2.0 with objectively focusing on children and parents’ perceptions about the frequency and the severity of the symptoms of EoE is very important for thoroughly and objectively evaluating the disease from the perspectives of both the patients and the parents. Since the first description of EoE in 2012 in Turkey, pediatric gastroenterologists and pediatric allergists have been increasingly recognizing EoE patients in their daily clinical practice (8). A study investigating validity and reliability of PEESv2.0 after translation and cultural adaptation of PEESv2.0 to Turkish language will be very useful for the sake of timely EoE diagnosis in Turkish patients and also for research purposes.

During the translation process, after forward and backward translations, assessment of content validity objectively on a point scale by 6 experts in the field provided very valuable contribution to the finalized version of T-PEESv2.0. Each item rated less than the full point WAS discussed with the investigators of the study and revised. The phrase in the frequency domain “almost never” had initially been translated as “Hemen hemen hiç” into Turkish. Later, it was revised as “Neredeyse hiç,” which had a similar meaning but was found more understandable by the experts. The first translation of “Bad” as “Kötü” was revised as “Şiddetli,” which was closer to the original meaning in the severity domain. Instead of “…than others”, “…than other children” was added to the question 11, 19, and 20 in PEESv2.0-parent by proxy, which made the statements more coherent in Turkish. All the revised versions ranked the full score by all 6 experts and no disagreements was observed. In the cognitive interview with the children and the parents, Question 17 was needed to be revised which made the meaning clearer from the patient/parent view. Maintenance of this perspective in T-PEESv2.0 was very important since the key distinction between the PEESv2.0 compared with the PEESv1.0 was the reflection of the second one the patient/parent view (3).

Turkish translation and language validation of PEESv2.0 generated T-PEESv2.0. The assessment of its validation and reliability was the second part of the study. Multicentrically, a cohort of patients with a clinicopathologic diagnosis of EoE who had been following up by pediatric allergy and gastroenterology clinics was confined. A healthy control group with similar characteristics was also generated. EoE is a disease with male predominance in both children and adults (6). Similarly, two-thirds of the study cohort were composed of male patients. The average age of EoE diagnosis in the pediatric population was typically between 6 and 10 years of age resembling to the median age of the study cohort which were around 9 (7).

This study showed that T-PEESv2.0 was a valid and reliable metric to assess EoE symptoms of patients/parents between ages of 2 and 18 years. T-PEESv2.0 was clearly discriminated both the frequency and the severity of the symptoms of EoE subjects from those of the healthy control group. All of the 20 items in Turkish translations of both metrics showed excellent internal consistency as a group. High index of agreement between repeated measurements of T-PEESv2.0 also demonstrated that it was highly reliable.

The authors did not determine construct validity by comparing the T-PEESv2.0 to another test that measure similar qualities. This may be a limitation of this study. However, a validated symptomatology score encompassing EoE symptomatology over the ages from 2 to 18 years has not existed in Turkish.

In conclusion, T-PEESv2.0 has been translated and culturally adapted to Turkish language. The study showed that T-PEESv2.0 has excellent validity and reliability with its both metrics and can be used for objective symptom assessment in pediatric EoE patients and also research purposes.
